# The chromosomal genome sequence of the marine leech, *Branchellion lobata *Moore, 1952 and its associated microbial metagenome sequences

**DOI:** 10.12688/wellcomeopenres.24186.1

**Published:** 2025-06-02

**Authors:** Shana K. Goffredi, Ralph Appy, José M. Martín-Durán, Graeme Oatley, Elizabeth Sinclair, Eerik Aunin, Noah Gettle, Camilla Santos, Michael Paulini, Haoyu Niu, Victoria McKenna, Rebecca O’Brien, Wellcome Sanger Institute Tree of Life Management, Samples and Laboratory Team, Wellcome Sanger Institute Scientific Operations: Sequencing Operations, Wellcome Sanger Institute Tree of Life Core Informatics Team, EBI Aquatic Symbiosis Genomics Data Portal Team, Aquatic Symbiosis Genomics Project Leadership

**Affiliations:** 1 Occidental College, Los Angeles, California, USA; 2 Cabrillo Marine Aquarium, San Pedro, California, USA; 3 Queen Mary University of London School of Biological and Behavioural Sciences, London, England, UK; 4 Tree of Life, Wellcome Sanger Institute, Hinxton, England, UK

**Keywords:** Branchellion lobata, marine leech, genome sequence, chromosomal, Hirudinida; microbial metagenome

## Abstract

We present a genome assembly from an individual *Branchellion lobata* (the marine leech; Annelida; Clitellata; Hirudinida; Piscicolidae). The genome sequence is 174.1 megabases in span. Most of the assembly is scaffolded into 17 chromosomal pseudomolecules. The mitochondrial genome has also been assembled and is 16.48 kilobases in length. The metagenome of *Branchellion lobata* was also assembled. Of ten binned metagenomes, three were classified as high-quality assembled genomes (MAGs).

## Species taxonomy

Eukaryota; Opisthokonta; Metazoa; Eumetazoa; Bilateria; Protostomia; Spiralia; Lophotrochozoa; Annelida; Clitellata; Hirudinea; Hirudinida; Oceanobdelliformes; Piscicolidae; *Branchellion*; *Branchellion lobata* Moore, 1952 (NCBI:txid375517).

## Background

Leeches are highly unusual annelids (class Clitellata; subclass Hirudinea) present in both terrestrial and aquatic habitats. They display a diversity of behaviours and nutritional strategies, ranging from parasitic blood-feeding to predatory non-blood-feeding. Marine leeches that prey on elasmobranchs and teleosts belong to the family Piscicolidae (order Rhynchobdellida), which comprises ~60 genera and 120 species ( Burreson, 2020; Mann, 1962; Sawyer, 1986). Compared to terrestrial leeches, little is known about marine leeches, likely due to their inaccessibility, seasonality, and non-viability in captivity ( Kearn, 2005). *Branchellion lobata* Moore 1952, is a species of marine leech found off the coasts of California, Chile, Costa Rica, Mexico, and Panama, that preys upon numerous elasmobranchs, including angel sharks, leopard sharks, bat rays, and butterfly rays ( Burreson, 2006; Moser & Anderson, 1977; Ruiz-Escobar & Oceguera-Figueroa, 2019). Due to the difficulty of sampling their nomadic elasmobranch hosts, there are gaps in our understanding of *Branchellion* broadly.

Blood feeders must contend with vitamin deficiencies (especially B vitamins), and red blood cells that are difficult to digest ( Toh *et al.*, 2010). To overcome these dietary hurdles, they often partner with internal bacteria that are believed to play an important role in counteracting the low digestibility and vitamin B deficiency, specifically. A study by Goffredi *et al.* (2023) showed that the *Branchellion* microbiome consisted of three main bacteria: *Vibrio* (~50% of the community), and undescribed gammaproteobacterial, which is also present in hatchlings, and an undescribed betaproteobacteria, the latter two each comprising ~16% of

## Genome sequence report for the host

**Figure 1.**
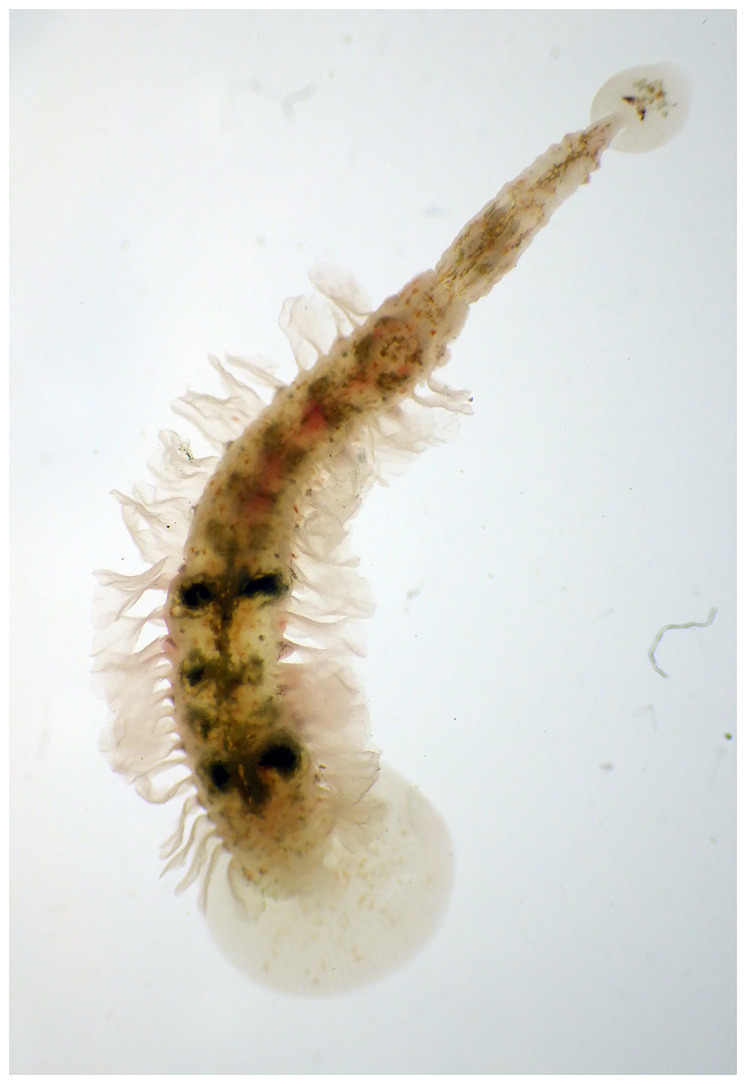
Photograph of a *Branchellion lobata* (not the specimen used for genome sequencing).

The genome was sequenced from an adult *Branchellion lobata* ( Figure 1) collected from Mugu Lagoon, Ventura County, California, USA (34.11, –119.13). A total of 201-fold coverage in Pacific Biosciences single-molecule HiFi long reads was generated. Primary assembly contigs were scaffolded with chromosome conformation Hi-C data. Manual assembly curation corrected 176 missing joins or mis-joins and removed 43 haplotypic duplications, reducing the assembly length by 1.45% and the scaffold number by 60.83%, and increasing the scaffold N50 by 8.41%.

**Figure 2.**
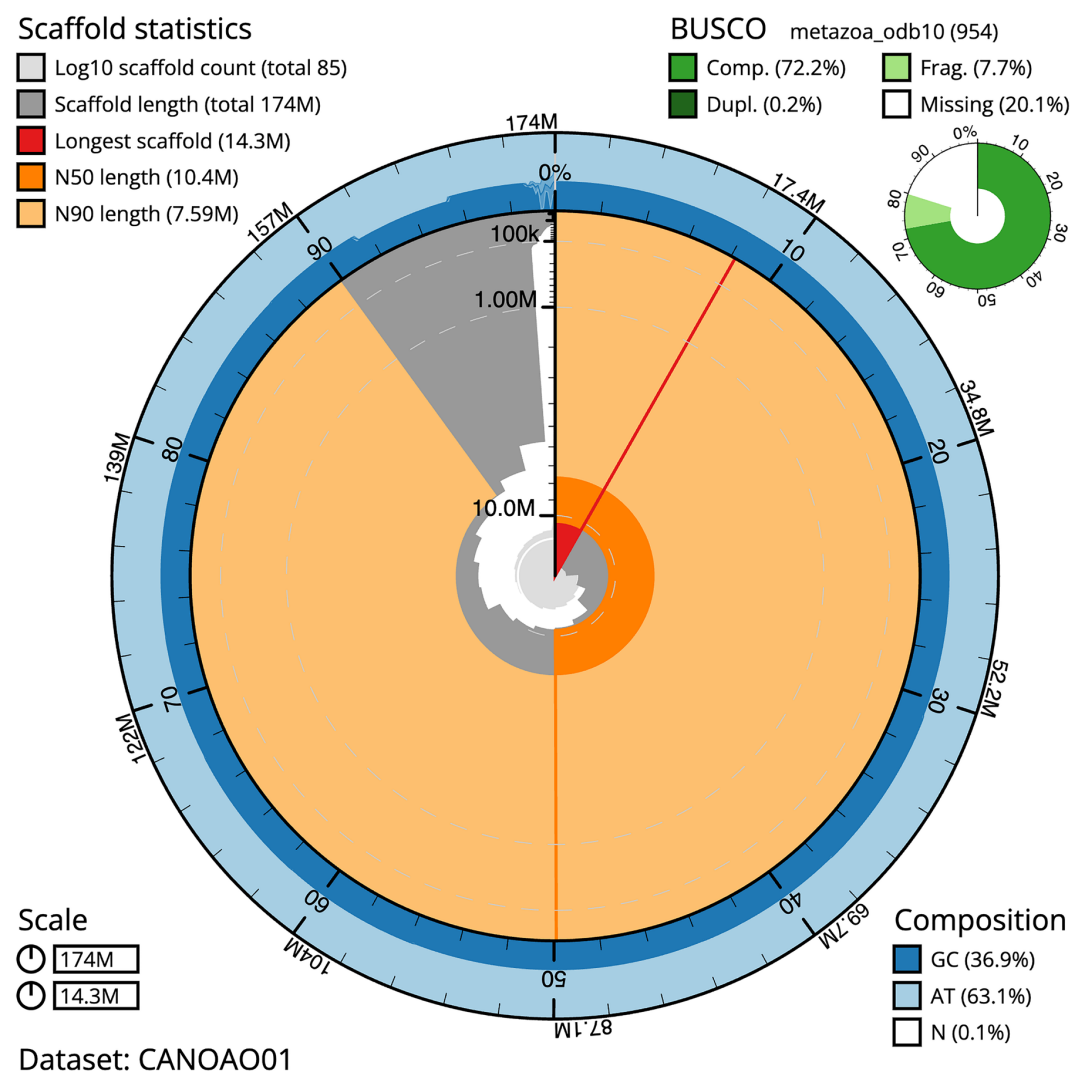
Genome assembly of *Branchellion lobata*, wcBraLoba9.1: metrics. The BlobToolKit Snailplot shows N50 metrics and BUSCO gene completeness. The main plot is divided into 1,000 size-ordered bins around the circumference with each bin representing 0.1% of the 174,144,220 bp assembly. The distribution of scaffold lengths is shown in dark grey with the plot radius scaled to the longest scaffold present in the assembly (14,345,173 bp, shown in red). Orange and pale-orange arcs show the N50 and N90 scaffold lengths (10,374,467 and 7,593,766 bp), respectively. The pale grey spiral shows the cumulative scaffold count on a log scale with white scale lines showing successive orders of magnitude. The blue and pale-blue area around the outside of the plot shows the distribution of GC, AT and N percentages in the same bins as the inner plot. A summary of complete, fragmented, duplicated and missing BUSCO genes in the metazoa_odb10 set is shown in the top right. An interactive version of this figure is available at https://blobtoolkit.genomehubs.org/view/Branchellion%20lobata/dataset/CANOAO01/snail.

**Figure 3.**
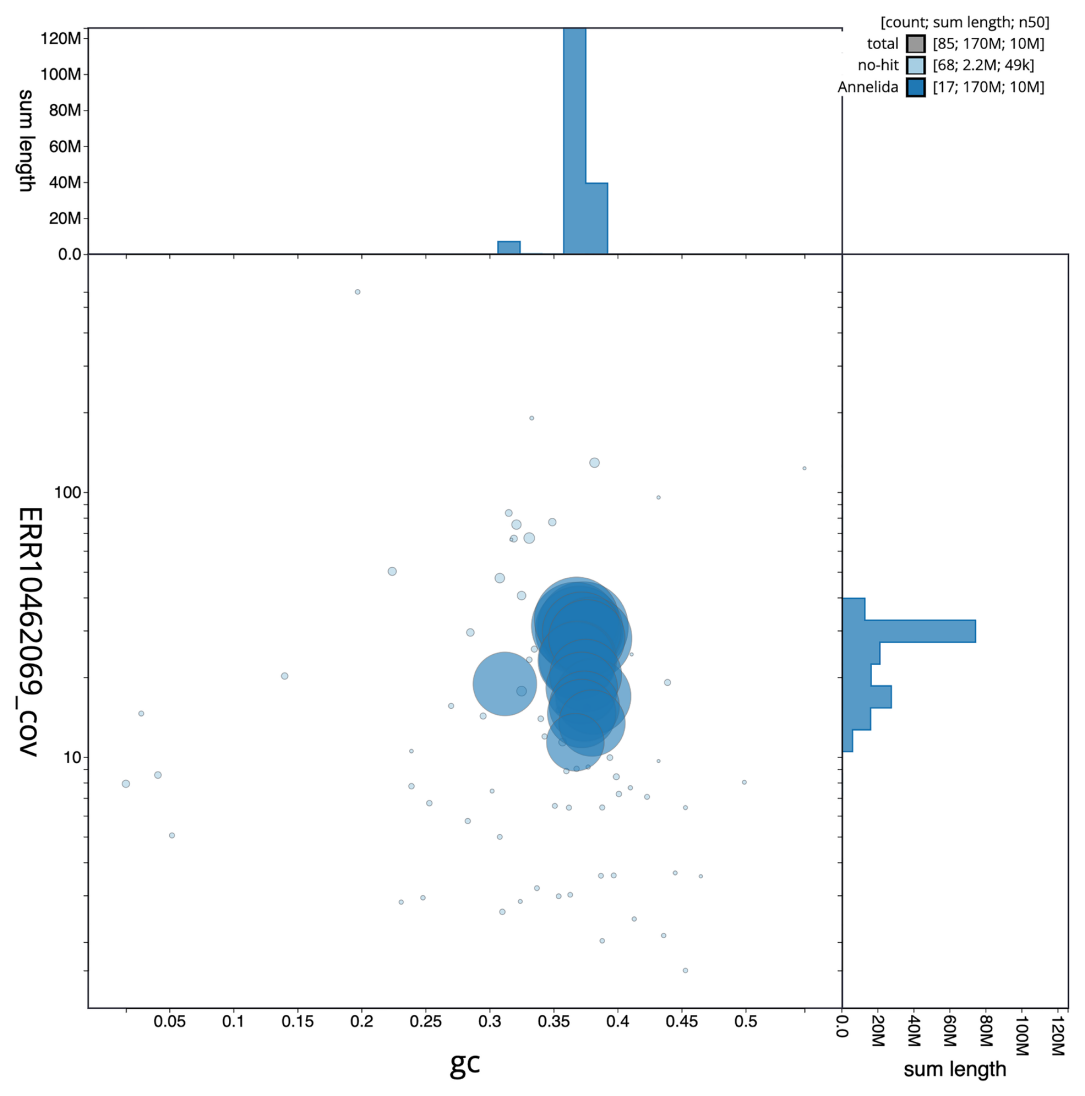
Genome assembly of *Branchellion lobata*, wcBraLoba9.1: BlobToolKit GC-coverage plot. Scaffolds are coloured by phylum. Circles are sized in proportion to scaffold length. Histograms show the distribution of scaffold length sum along each axis. An interactive version of this figure is available at https://blobtoolkit.genomehubs.org/view/Branchellion%20lobata/dataset/CANOAO01/blob.

**Figure 4.**
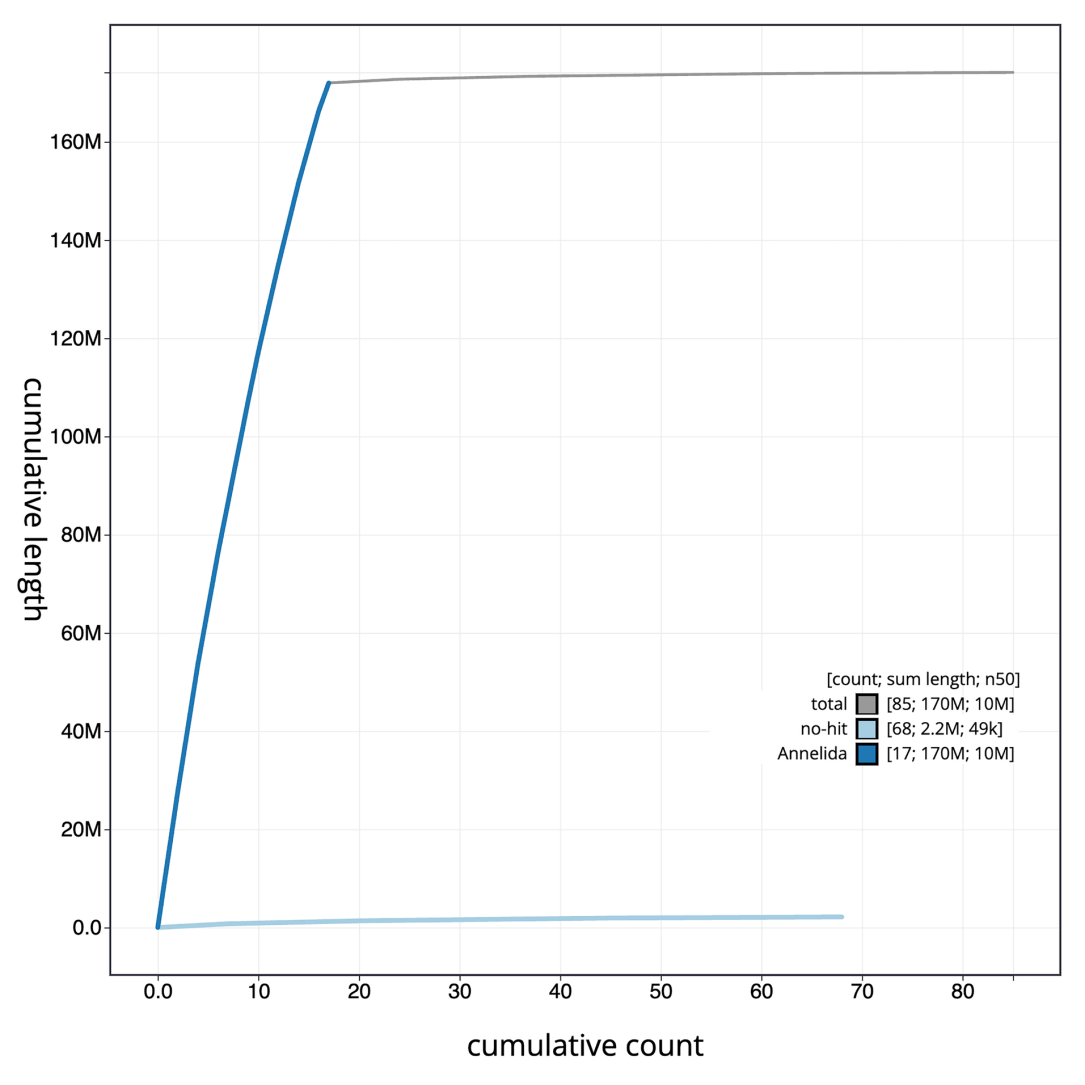
Genome assembly of *Branchellion lobata*, wcBraLoba9.1: BlobToolKit cumulative sequence plot. The grey line shows cumulative length for all scaffolds. Coloured lines show cumulative lengths of scaffolds assigned to each phylum using the buscogenes taxrule. An interactive version of this figure is available at https://blobtoolkit.genomehubs.org/view/Branchellion%20lobata/dataset/CANOAO01/cumulative.

**Figure 5.**
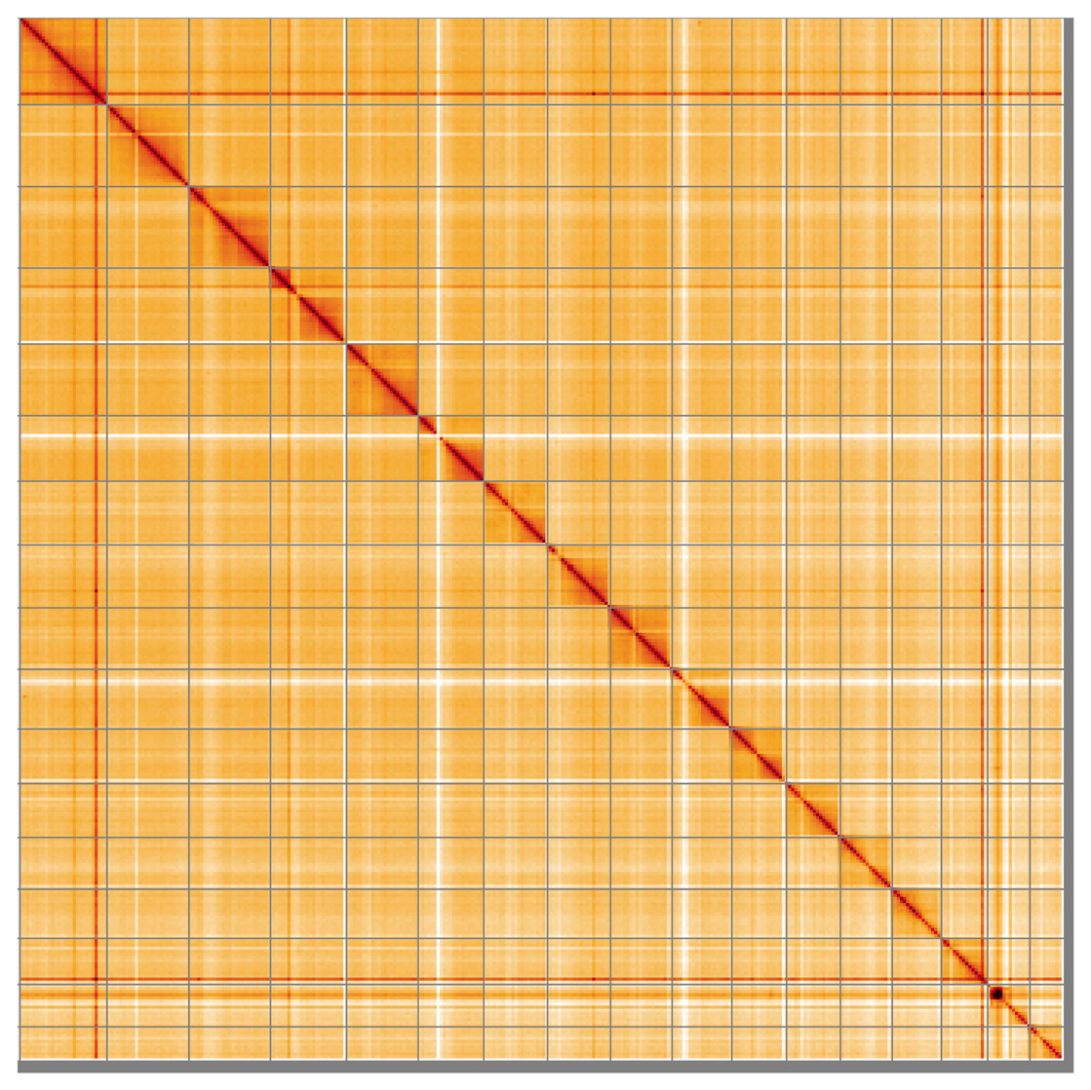
Genome assembly of *Branchellion lobata*, wcBraLoba9.1: Hi-C contact map of the wcBraLoba9.1 assembly, visualised using HiGlass. Chromosomes are shown in order of size from left to right and top to bottom. An interactive version of this figure may be viewed at https://genome-note-higlass.tol.sanger.ac.uk/l/?d=OLHyD15BQFuW1A4iz0VWpw.

The final assembly has a total length of 174.1 Mb in 84 sequence scaffolds with a scaffold N50 of 10.4 Mb ( Table 1). The snail plot in Figure 2 provides a summary of the assembly statistics, while the distribution of assembly scaffolds on GC proportion and coverage is shown in Figure 3. The cumulative assembly plot in Figure 4 shows curves for subsets of scaffolds assigned to different phyla. Most (98.81%) of the assembly sequence was assigned to 17 chromosomal-level scaffolds. Chromosome-scale scaffolds confirmed by the Hi-C data are named in order of size ( Figure 5; Table 2). While not fully phased, the assembly deposited is of one haplotype. Contigs corresponding to the second haplotype have also been deposited. The mitochondrial genome was also assembled and can be found as a contig within the multifasta file of the genome submission.

**Table 1.** Genome data for *Branchellion lobata*, wcBraLoba9.1.

Project accession data	
Assembly identifier	wcBraLoba9.1
Species	*Branchellion lobata*
Specimen	wcBraLoba9
NCBI taxonomy ID	375517
BioProject	PRJEB57262
BioSample ID	Genome sequencing: SAMEA11604974 Hi-C scaffolding: SAMEA11604973 RNA sequencing: SAMEA11604970
Isolate information	wcBraLoba9: whole organism (genome sequence) wcBraLoba8: whole organism (Hi-C sequencing) wcBraLoba5: whole organism (RNA sequencing)
Assembly metrics	
Consensus quality (QV)	47.8
*k*-mer completeness	99.95%
BUSCO *	C:72.5%[S:72.3%,D:0.2%], F:7.4%,M:20.1%,n:954
Percentage of assembly mapped to chromosomes	98.81%
Organelles	Mitochondrial genome: 16.48 kb
PacificBiosciences Sequel IIe	ERR10462068, ERR10462069, ERR10462070
Hi-C Illumina	ERR10466804
PolyA RNA-Seq Illumina	ERR12708735
Genome assembly	
Assembly accession	GCA_947562095.1
*Accession of alternate haplotype*	GCA_947563305.1
Span (Mb)	174.1
Number of contigs	720
Contig N50 length (Mb)	0.5
Number of scaffolds	84
Scaffold N50 length (Mb)	10.4
Longest scaffold (Mb)	14.37

* BUSCO scores based on the metazoa_odb10 BUSCO set using version 5.3.2. C = complete [S = single copy, D = duplicated], F = fragmented, M = missing, n = number of orthologues in comparison. A full set of BUSCO scores is available at https://blobtoolkit.genomehubs.org/view/Branchellion lobata/dataset/CANOAO01/busco.

**Table 2.** Chromosomal pseudomolecules in the genome assembly of *Branchellion lobata*, wcBraLoba9.

INSDC accession	Name	Length (Mb)	GC%
OX387246.1	1	14.35	37.5
OX387247.1	2	13.48	37.0
OX387248.1	3	13.42	36.5
OX387249.1	4	12.52	37.0
OX387250.1	5	11.81	38.0
OX387251.1	6	10.81	37.0
OX387252.1	7	10.49	37.0
OX387253.1	8	10.37	37.0
OX387254.1	9	10.13	37.5
OX387255.1	10	9.84	38.0
OX387256.1	11	9.01	37.5
OX387257.1	12	8.88	37.0
OX387258.1	13	8.52	37.5
OX387259.1	14	8.11	37.0
OX387260.1	15	7.59	38.0
OX387261.1	16	6.98	31.0
OX387262.1	17	5.67	36.5
OX387263.1	MT	0.02	20.0

The estimated Quality Value (QV) of the final assembly is 47.8 with *k*-mer completeness of 99.95%, and the assembly has a BUSCO v completeness of 72.5% (single = 72.3%, duplicated = 0.2%), using the metazoa_odb10 reference set ( *n* = 954).

## Metagenome report

**Figure 6.**
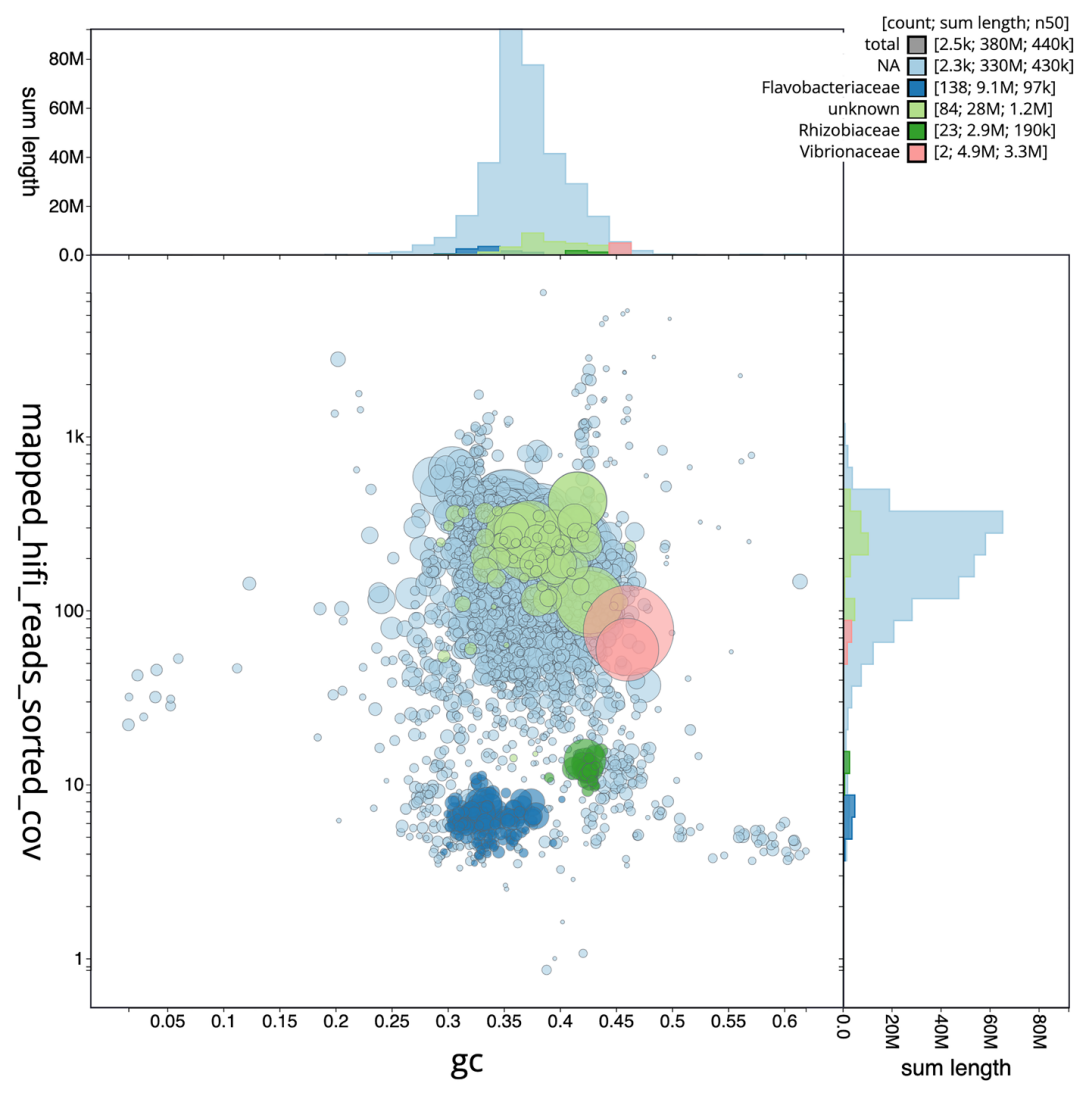
Blob plot of base coverage in mapped against GC proportion for sequences in the metagenome of the *Branchellion lobata* sample. Sequences are coloured by family. Circles are sized in proportion to sequence length on a square-root scale, ranging from 1,608 to 3,301,098. Histograms show the distribution of sequence length sum along each axis. An interactive version of this figure may be viewed here.

Ten binned genomes were generated from the metagenome assembly ( Figure 6), of which three were classified as high-quality metagenome assembled genomes (MAG) (see methods). The completeness values for these assemblies range from approximately 36% to 99% with contamination below 5%. For details on binned genomes see Table 3.

**Table 3.** Quality metrics and taxonomic assignments of the MAGs.

NCBI taxon	Taxid	GTDB taxonomy	Quality	Size (bp)	Contigs	Circular	Mean coverage	Completeness (%)	Contamination (%)
Rhizobiaceae bacterium	271151	g__JAALLB01	High	2,997,465	23	No	83.83	91.08	4.32
uncultured *Mesonia* sp.	399731	g__Mesonia	High	2,787,421	29	No	93.98	92.54	0.19
*Vibrio sinaloensis*	379097	s__Vibrio sinaloensis	High	4,973,486	2	Partial	61.73	99.36	4.52
Gammaproteobacteria bacterium	86473	f__JAAOII01	Medium	1,857,420	1	No	83.39	86.44	3.27
Burkholderiales bacterium	208544	o__Burkholderiales	Medium	23,459,930	80	Partial	100.72	89.25	2
uncultured Aureibaculum sp.	2911477	g__Aureibaculum	Medium	3,158,360	29	No	97.02	84.94	0.13
Mesoflavibacter zeaxanthinifaciens	393060	s__Mesoflavibacter zeaxanthinifaciens	Medium	2,138,924	43	No	91.76	66.11	0
Burkholderiales bacterium	208544	o__Burkholderiales	Medium	1,393,589	1	Yes	94.27	96.34	0
Bizionia echini	649333	s__Algorimicrobium echini	Low	1,216,795	37	No	93.03	36.25	0
Gammaproteobacteria bacterium	86473	f__JAAOII01	Medium	1,843,069	2	No	88.25	86.44	1.23

## Methods

### Sample acquisition

*Branchellion lobata* specimens were collected from the Pacific round ray ( *Urobatis halleri*) from Mugu lagoon salt marsh (34°06'19.5"N 119°07'33.2"W; Ventura County, California) on Sept 23, 2021. The host animal was collected by beach seine under specific use permit ID: S-200810003-20163-001 to Ralph Appy). Leeches were removed and preserved on dry ice, before storing at –80 °C. Specimen ID QMOUL0000035 (ToLID wcBraLoba9) was used for long read PacBio sequencing, specimen ID QMOUL0000034 (ToLID wcBraLoba8) was used for Hi-C sequencing and specimen ID QMOUL0000031 (ToLID wcBraLoba5) was used for RNA sequencing.

### Nucleic acid extraction

Protocols developed by the Wellcome Sanger Institute (WSI) Tree of Life laboratory are publicly available on protocols.io ( Howard *et al.*, 2025). The workflow for high molecular weight (HMW) DNA extraction includes a sequence of procedures: sample preparation; sample homogenisation, DNA extraction, fragmentation, and clean-up. In sample preparation, the wcBraLoba9 sample was weighed and dissected on dry ice ( Jay *et al.*, 2023). Tissue from the whole organism was homogenised using a PowerMasher II tissue disruptor ( Denton *et al.*, 2023). HMW DNA was extracted using the Manual MagAttract v1 protocol ( Strickland *et al.*, 2023b). DNA was sheared into an average fragment size of 12–20 kb in a Megaruptor 3 system with speed setting 30 ( Todorovic *et al.*, 2023). Sheared DNA was purified by solid-phase reversible immobilisation ( Strickland *et al.*, 2023a), using AMPure PB beads to eliminate shorter fragments and concentrate the DNA. The concentration of the sheared and purified DNA was assessed using a Nanodrop spectrophotometer and Qubit Fluorometer and Qubit dsDNA High Sensitivity Assay kit. Fragment size distribution was evaluated by running the sample on the FemtoPulse system.

RNA was extracted from whole organism tissue of wcBraLoba5 in the Tree of Life Laboratory at the WSI using the RNA Extraction: Automated MagMax™ *mir*Vana protocol ( do Amaral *et al.*, 2023). The RNA concentration was assessed using a Nanodrop spectrophotometer and a Qubit Fluorometer using the Qubit RNA Broad-Range Assay kit. Analysis of the integrity of the RNA was done using the Agilent RNA 6000 Pico Kit and Eukaryotic Total RNA assay.

### Sequencing

Pacific Biosciences HiFi circular consensus DNA sequencing libraries were constructed according to the manufacturers’ instructions. Poly(A) RNA-Seq libraries were constructed using the NEB Ultra II RNA Library Prep kit. DNA and RNA sequencing was performed by the Scientific Operations core at the WSI on Pacific Biosciences Sequel IIe (HiFi) and Illumina NovaSeq 6000 (RNA-Seq) instruments. Hi-C data were also generated from whole organism tissue of wcBraLoba8 using the Arima2 kit and sequenced on the Illumina NovaSeq 6000 instrument.

### Genome assembly and curation

Assembly was carried out with Hifiasm ( Cheng *et al.*, 2021) and haplotypic duplication was identified and removed with purge_dups ( Guan *et al.*, 2020). The assembly was then scaffolded with Hi-C data ( Rao *et al.*, 2014) using YaHS ( Zhou *et al.*, 2023). The assembly was checked for contamination and corrected using the gEVAL system ( Chow *et al.*, 2016) as described previously ( Howe *et al.*, 2021). Manual curation was performed using gEVAL, HiGlass ( Kerpedjiev *et al.*, 2018) and Pretext ( Harry, 2022). The mitochondrial genome was assembled using MitoHiFi ( Uliano-Silva *et al.*, 2023), which runs MitoFinder ( Allio *et al.*, 2020) and uses these annotations to select the final mitochondrial contig and to ensure the general quality of the sequence.

The metagenome assembly was generated using Hifiasm ( Cheng *et al.*, 2021) and binned using MetaBAT2 ( Kang *et al.*, 2019), MaxBin ( Wu *et al.*, 2014), bin3C ( DeMaere & Darling, 2019), and MetaTOR. The resulting bin sets of each binning algorithm were individually optimised and then collectively refined using DAS Tool ( Sieber *et al.*, 2018). PROKKA ( Seemann, 2014) was used to identify tRNAs and rRNAs in each bin, CheckM ( Parks *et al.*, 2015) (checkM_DB release 2015-01-16) was used to assess bin completeness/contamination, and GTDB-TK ( Chaumeil *et al.*, 2022) (GTDB release 214) was used to taxonomically classify bins. Taxonomic replicate bins were identified using dRep ( Olm *et al*., 2017) with default settings (95% ANI threshold). The final bin set was filtered for bacteria and archaea. All bins were assessed for quality and categorised as metagenome-assembled genomes (MAGs) if they met the following criteria: contamination ≤ 5%, presence of 5S, 16S, and 23S rRNA genes, at least 18 unique tRNAs, and either ≥ 90% completeness or ≥ 50% completeness with fully circularised chromosomes. Bins that did not meet these thresholds, or were identified as taxonomic replicates of MAGs, were retained as ‘binned metagenomes’ provided they had ≥ 50% completeness and ≤ 10% contamination. Software tool versions and sources are given in Table 4.

**Table 4.** Software tools: versions and sources.

Software tool	Version	Source
bin3C	version 0.3.3	https://github.com/cerebis/bin3C
BlobToolKit	4.2.1	https://github.com/blobtoolkit/blobtoolkit
BUSCO	5.3.2	https://gitlab.com/ezlab/busco
bwa-mem2	2.2.1	https://github.com/bwa-mem2/bwa-mem2
CheckM	1.2.1	https://github.com/Ecogenomics/CheckM
DAS Tool	1.1.5	https://github.com/cmks/DAS_Tool
dRep	3.4.0	https://github.com/MrOlm/drep
FastK	427104ea91c78c3b8b8b49f1a7d6bbeaa869ba1c	https://github.com/thegenemyers/FASTK
gEVAL	N/A	https://geval.org.uk/
GTDB-TK	2.3.2	https://github.com/Ecogenomics/GTDBTk
Hifiasm	0.16.1	https://github.com/chhylp123/hifiasm
HiGlass	1.11.6	https://github.com/higlass/higlass
MaxBin	version 2.7	https://github.com/jtamames/SqueezeMeta/tree/master/bin/MaxBin
MerquryFK	d00d98157618f4e8d1a9190026b19b471055b22e	https://github.com/thegenemyers/MERQURY.FK
MetaBAT2	version 2.15-15-gd6ea400	https://bitbucket.org/berkeleylab/metabat/src/master/
MetaTOR	-	https://github.com/koszullab/metaTOR
MitoHiFi	2	https://github.com/marcelauliano/MitoHiFi
PretextView	0.2	https://github.com/wtsi-hpag/PretextView
PROKKA	1.14.5	https://github.com/vdejager/prokka
purge_dups	1.2.3	https://github.com/dfguan/purge_dups
YaHS	1.1a.2	https://github.com/c-zhou/yahs

### Assembly quality assessment

The Merqury.FK tool ( Rhie *et al.*, 2020), run in a Singularity container ( Kurtzer *et al.*, 2017), was used to evaluate *k*-mer completeness and assembly quality for the primary and alternate haplotypes using the *k*-mer databases ( *k* = 31) that were computed prior to genome assembly. The analysis outputs included assembly QV scores and completeness statistics.

A Hi-C contact map was produced for the final version of the assembly. The Hi-C reads were aligned using bwa-mem2 ( Vasimuddin *et al.*, 2019) and the alignment files were combined using SAMtools ( Danecek *et al.*, 2021). The Hi-C alignments were converted into a contact map using BEDTools ( Quinlan & Hall, 2010) and the Cooler tool suite ( Abdennur & Mirny, 2020). The contact map is visualised in HiGlass ( Kerpedjiev *et al.*, 2018).

The genome was also analysed within the BlobToolKit environment ( Challis *et al.*, 2020) and BUSCO scores ( Manni *et al.*, 2021) were calculated.

Table 4 contains a list of relevant software tool versions and sources.

### Wellcome Sanger Institute – Legal and Governance

The materials that have contributed to this genome note have been supplied by a Tree of Life collaborator. The Wellcome Sanger Institute employs a process whereby due diligence is carried out proportionate to the nature of the materials themselves, and the circumstances under which they have been/are to be collected and provided for use. The purpose of this is to address and mitigate any potential legal and/or ethical implications of receipt and use of the materials as part of the research project, and to ensure that in doing so we align with best practice wherever possible. The overarching areas of consideration are:

•     Ethical review of provenance and sourcing of the material

•     Legality of collection, transfer and use (national and international)

Each transfer of samples is undertaken according to a Research Collaboration Agreement or Material Transfer Agreement entered into by the Tree of Life collaborator, Genome Research Limited (operating as the Wellcome Sanger Institute) and in some circumstances other Tree of Life collaborators.

## Data Availability

European Nucleotide Archive: *Branchellion lobata* (marine leech). Accession number PRJEB57262; https://identifiers.org/ena.embl/PRJEB57262. The genome sequence is released openly for reuse. The *Branchellion lobata* genome sequencing initiative is part of the Aquatics Symbiosis Genomics (ASG) project. All raw sequence data and the assembly have been deposited in INSDC databases. Raw data and assembly accession identifiers are reported in Table 1.

## Author information

Members of the Wellcome Sanger Institute Tree of Life Management, Samples and Laboratory Team are listed here: https://doi.org/10.5281/zenodo.10066175.

Members of the Wellcome Sanger Institute Scientific Operations: Sequencing Operations are listed here: https://doi.org/10.5281/zenodo.10043364.

Members of the Wellcome Sanger Institute Tree of Life Core Informatics team are listed here: https://doi.org/10.5281/zenodo.14870789.

Members of the European Bioinformatics Institute ASG Data Portal team are listed here: https://doi.org//10.5281/zenodo.10076466.

Members of the Wellcome Sanger Institute/Aquatic Symbiosis Genomics Project Leadership are listed here: https://doi.org/10.5281/zenodo.10184833.
