# Supplementary material for: The chromosomal genome sequence of the marine leech, Branchellion lobata Moore, 1952 and its associated microbial metagenome sequences
Source: Wellcome Open Res. 2025 Jun 2;10:304. [Version 1] doi: 10.12688/wellcomeopenres.24186.1 (PMC12326168; doi:10.12688/wellcomeopenres.24186.1)
Supplement: Peer review history

# Reviewer report: Reviewer response for version 1

**DOI:** 10.21956/wellcomeopenres.26679.r125791

**Published:** 2025-08-05

**Authors:** David A WEISBLAT

**Affiliations:** 1 University of California, Berkeley, California, USA

*Beyond the general interest in accumulating as inclusive a set of genome sequences as possible, sequencing genomes of leeches and other ciltellate annelids is of interest because of the extreme acceleration of genome rearrangements within this group of annelids. A dense sampling of clitellate genomes is needed to reconstruct the history of these genome rearrangements and eventually understand the underlying cause(s).*

*I do not have the expertise to judge the technical aspects of this work, but it appears to be carefully done. It would be nice to see how the inferred number of 17 chromosomes compares to an cytologically determined karyotype.*

*My only substantive comment is in reference to a statement in the Background "* *Marine leeches that prey on elasmobranchs and teleosts belong to the family Piscicolidae (order Rhynchobdellida)....".  My understanding is that Rhynchobdellida, though still commonly recognized, may not be a monophyletic grouping. Traditionally, rhynchobdellid leeches include both glossiphoniid and piscicolid leeches (proboscis-bearing species laying large yolky eggs), as distinct from a clade containing erpobdellid and hirudinid species (jawed instead of proboscis-bearing, and laying small embryos in albumen-filled cocoons). However, more recent molecular phylogenies based on extensive nuclear gene data (including a collaboration in which I am participating:*

**Schultz D, et al., 2024 [Ref 1] *)* *supports a tree in which piscicolids are sister to the group of erpobdellids and hirudinids, with glossiphoniids as an outgroup to all of these. This revised tree is significant for leech biologists because it supports the hypothesis that ancestral leeches had probosces and laid large yolky eggs and pushes the evolution of jawed leeches with small embryos to more recent times.*

*I note that the sentence in question is also at odds with the species taxonomy given in the article as well.*

Are sufficient details of methods and materials provided to allow replication by others?

Yes

Is the rationale for creating the dataset(s) clearly described?

Yes

Are the datasets clearly presented in a useable and accessible format?

Yes

Are the protocols appropriate and is the work technically sound?

Yes

Reviewer Expertise:

NA

I confirm that I have read this submission and believe that I have an appropriate level of expertise to confirm that it is of an acceptable scientific standard.

# Reviewer report: Reviewer response for version 1

**DOI:** 10.21956/wellcomeopenres.26679.r125785

**Published:** 2025-07-24

**Authors:** Wanchang Zhang

**Affiliations:** 1 Nanchang University, Nanchang, China

The authors present the genome assembly of a marine leech, *Branchellion lobata*, which feeds on teleost hosts. The organism is interesting. The genome assembly of *Branchellion lobata* is valuable for future research. The authors clearly declared the results and methods of the genome and metagenome assembly of the leech. The manuscript is clearly written; I believe the manuscript could be indexed after addressing some minor comments.

 It seems that the authors didn’t conduct genome annotation to the leech assembly, how many genes are annotated along the genome? Once a genome was published, the genome annotation is also important to perform downstream functional analyses.

 A comparable chromosome Syteny analysis between this organism and other close leech organism could be carried out to assess the genome completeness.

 Figure 1, a scale bar should be present.

Are sufficient details of methods and materials provided to allow replication by others?

Yes

Is the rationale for creating the dataset(s) clearly described?

Yes

Are the datasets clearly presented in a useable and accessible format?

Partly

Are the protocols appropriate and is the work technically sound?

Yes

Reviewer Expertise:

Fish genetics

I confirm that I have read this submission and believe that I have an appropriate level of expertise to confirm that it is of an acceptable scientific standard, however I have significant reservations, as outlined above.

# Reviewer report: Reviewer response for version 1

**DOI:** 10.21956/wellcomeopenres.26679.r125053

**Published:** 2025-06-27

**Authors:** Christian Müller

**Affiliations:** 1 University of Greifswald, Greifswald, Germany

**Manuskript-ID**: 24186          

**Journal**:               Wellcome Open Research

**Title:**                     The chromosomal genome sequence of the marine leech, *Branchellion lobata* Moore, 1952 and its associated microbial metagenome sequences.

**Authors**:              Goffredi SK, Appy R, Martín-Durán JM, Oatley G, Sinclair E, Aunin E, Gettle N, Santos C, Paulini M, Niu H, McKenna V, O’Brien R

**Short Summary**

 The author(s) submitted a manuscript that describes the generation, assembly and quality analysis of the almost complete chromosomal genome and the complete mitochondrial genome of *Branchellion lobata*, a marine leech that feeds on cartilaginous fishes. In addition, the metagenome of *Branchellion lobata* was determined as well, including the genomes of the three most abundant bacterial species.  

**General Impression**

 The manuscript is part of the Tree of Life project in general and the Aquatic Symbiosis Genomics Project in detail. It addresses a topic that is in the first instance of interest mainly for biologist and ecologists, but beyond that also for a broad audience as it helps to shed light on the diversity of life. The manuscript is in line with a previous publication on the complete genome of *Piscicola geometra*, a limnic fish leech ( **Doe et al. 2023**), and follows in style and content comparable genome papers that were published in Wellcome Open Research.  

 I fully appreciate the efforts of the authors, and I have no doubts that all experiments and analyses were performed with accuracy. The manuscript is well written; the results and data are clearly presented. In that context I strongly recommend its indexing. There are only a few minor remarks that need to be addressed.

**Minor remarks:**

 1) The last sentence of the Background chapter is incomplete ("the latter two each comprising ~16% of "). Of what?   

 2) Figure 1: please indicate the source of the image

 3) The manuscript lacks any information on the sources of the lab equipment, the reagents and kits that were used.

 4) The manuscript lacks any further analyses like the identification of putative anticoagulants. At least a short comparison with the genome of *Piscicola geometra* would, in my opinion, significantly enhance the strength of the manuscript. Please consider.

Are sufficient details of methods and materials provided to allow replication by others?

Partly

Is the rationale for creating the dataset(s) clearly described?

Yes

Are the datasets clearly presented in a useable and accessible format?

Yes

Are the protocols appropriate and is the work technically sound?

Yes

Reviewer Expertise:

animal physiology

I confirm that I have read this submission and believe that I have an appropriate level of expertise to confirm that it is of an acceptable scientific standard, however I have significant reservations, as outlined above.

## References

[1] : The genome sequence of the fish leech, Piscicola geometra (Linnaeus, 1761). *Wellcome Open Research* .2023;8: 10.12688/wellcomeopenres.19488.1 10.12688/wellcomeopenres.19488.1 PMC1113415038813549
